# Comparison of standard and modified human landing catching techniques for blackfly collection

**DOI:** 10.1093/inthealth/ihad066

**Published:** 2023-08-25

**Authors:** Kareen Atekem, Philippe Nwane, Rogers Nditanchou, Anita Jeyam, Aude Wilhelm, Richard Selby, Louise Hamill, Elena Schmidt, Ruth Dixon, Daniel Boakye

**Affiliations:** Department of Entomology, Center for Infectious Disease Dynamics, Pennsylvania State University, University Park, PA, USA; Sightsavers – Yaoundé, Cameroon; Haywards Heath, UK; Centre for Research on Filariasis and Other Tropical Diseases, Yaoundé, Cameroon; University of Yaoundé I, Cameroon; Sightsavers – Yaoundé, Cameroon; Haywards Heath, UK; Sightsavers – Yaoundé, Cameroon; Haywards Heath, UK; Sightsavers – Yaoundé, Cameroon; Haywards Heath, UK; Sightsavers – Yaoundé, Cameroon; Haywards Heath, UK; Sightsavers – Yaoundé, Cameroon; Haywards Heath, UK; Sightsavers – Yaoundé, Cameroon; Haywards Heath, UK; Sightsavers – Yaoundé, Cameroon; Haywards Heath, UK; Parasitology Department, Noguchi Memorial Institute for Medical Research, University of Ghana, Legon, Accra; End Fund, New York, NY, USA

**Keywords:** blackfly, elimination, entomological evaluation, human landing collection, onchocerciasis, transmission

## Abstract

**Background:**

Human landing catches (HLCs) are required to collect blackflies for entomological evaluation to verify onchocerciasis elimination. However, there are ethical concerns regarding exposure of vector collectors to infectious blackflies and safer alternative methods are needed. This study evaluated a modified HLC technique where collectors wore coloured trousers (blue, black or blue-black), protecting them from bites during fly collection, and their performance was compared with the standard.

**Methods:**

The study was conducted in Makouopsap, Cameroon, in the Massangam health district for 4 months. Four collector pairs—one ‘standard’ (bare-legged) and three modified—were placed 50 m apart along known breeding sites on the Mbam and Nja Rivers. Collections were performed from 07:00 to 17:00 h, 4 d/month. Hourly rates of flies caught were analysed using a negative binomial generalised linear model to explore associations between flies caught and collection techniques and seasons.

**Results:**

Overall, 17 246 blackflies were caught. There was no significant statistical difference in the number of blackflies and parous flies caught between black trousers and the standard. Thus there is a strong indication that wearing black trousers is a viable non-inferior alternative to the standard HLC.

**Conclusions:**

Further studies are needed to confirm generalisability in different ecozones and transmission environments and among different blackfly species.

## Introduction

Female blackflies of the genus *Simulium* transmit *Onchocerca volvulus* (OV), the parasite responsible for human onchocerciasis (river blindness). This disease causes skin rashes and lesions, intense and debilitating itching and skin depigmentation. When the microfilariae (mf) migrate to the eye, they can cause ocular lesions that may subsequently result in blindness if not treated.^1^ Blackflies lay their eggs on substrates partially submerged in fast-flowing water, the eggs develop into larvae and then pupae, from which emerge adult flies that live in the terrestrial environment. Plant sugars are the main nutritional resource for blackflies, but females require a blood meal for viable egg development and are generally anthropophilic. During a blood meal, blackflies may either ingest OV mf or transmit infective-stage larvae (L3) into the skin of the human host. Thus, monitoring the infection in blackflies is an important component for evaluating the impact of interventions.^2,3^

Following the paradigm shift from onchocerciasis control to elimination of transmission, demonstrated by successful interruption of transmission in some African foci,^4–7^ countries are working towards achieving elimination targets by 2030.^8^ This necessitates the development of new tools and strategies to safely monitor disease transmission through xenomonitoring.^8,9^ The World Health Organization (WHO) guidelines for verification of onchocerciasis elimination place emphasis on entomological evaluations,^10,11^ requiring the collection of large numbers of anthropophilic blackflies (approximately 6000 blackflies). Human landing catch (HLC) is regarded as the gold standard tool for sampling host-seeking blackflies. In the framework of the evaluation of onchocerciasis transmission, this technique is used to collect female blackflies to assess entomological transmission indices and the efficacy of vector control measures.^10,12^

The HLC approach involves a volunteer sitting with their lower legs exposed, diligently collecting flies as they land on their exposed legs, before blood feeding starts. This technique of fly collection is mainly based on visual, olfactory and even thermal cues emitted by humans that attract the vectors.^13^ Ethically, the HLC technique is suboptimal, as collectors are exposed to potentially infective insect bites from the vectors intended for collection and others present in the ecosystem.^3,14,15^

The WHO acknowledges a need to develop new sampling techniques to replace HLC for the collection of blackflies.^16^ The choice of alternative methods for the collection of arthropod vectors of disease must consider the effectiveness in reproducing the vital elements that attract blackflies to humans. This mimicry is critical to the identification of anthropophilic species and effective evaluation of transmission dynamics.^17^ Thus, any alternative to HLC should collect appropriate numbers of the same vector populations, with a matching age structure (parity rates) as those biting humans. This means flies need to be in a condition that enables age identifying structures to be determined,^13^ at least for the validation of any new technique in comparison to the gold-standard HLC, posing challenges.

Various techniques have been explored as candidates for replacement of HLC.^18–20^ Unfortunately, proposed alternative techniques have demonstrated conflicting success rates depending on geographic locations, vector species and the type of baits used.^3,21^ Some techniques, such as the Esperanza Window Trap (EWT), have demonstrated great potential compared with other traps. But collections have not been reliable and consistent across geographic locations. While traps present a considerable logistical advantage, as two people rotating hourly can simultaneously operate multiple traps, no method has been validated to replace HLC.

Collection of large numbers of flies using HLC can be achieved by positioning vector collection sites around highly productive breeding sites, increasing the number of collectors and collecting throughout the transmission season. But the challenge of exposing vector collectors to infection during fly collection remains. This study aimed to evaluate the performance of a modified HLC technique through volunteers wearing protective clothing of colours known to be attractive to blackflies^21^ to prevent exposure to infective bites. In this article we will present the findings of preliminary testing performed in Cameroon and discuss the implications for replacement of traditional HLC.

## Methods

### Study area and period

**Figure 1.**
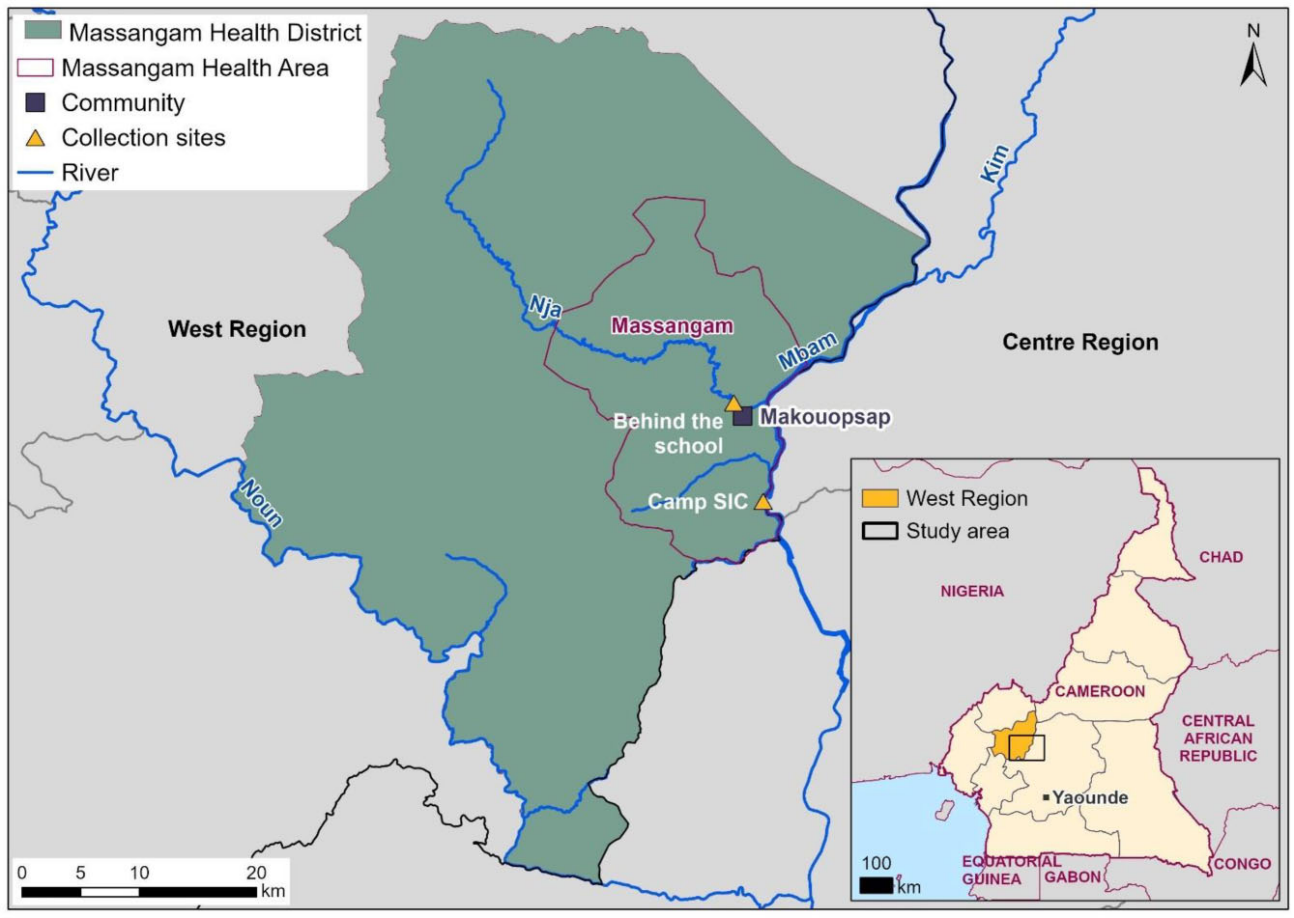
Map showing the study area and collection points. The map is from open-source maps retrieved on Diva-GIS (www.diva-gis.org/gdata). Rivers downloaded from HydroShed (https://www.hydrosheds.org/products/gloric). Geographical coordinates of communities and collection sites were collected using the Global Positioning System.

The study was conducted at Makouopsap in the Massangam Health District (HD), located in the West Region of Cameroon. The study area lies between 5°00′00′′ N and 5°35′00′′ N and 10°45′00′′ E to 11°15′00′′ E, a total area of 2216 km^2^ (Figure 1). The Massangam HD has a known high prevalence of onchocerciasis, with persistent transmission despite over two decades of ivermectin (IVM) mass drug administration (MDA) to afflicted populations.^22^

This area harbours two main rivers, the Mbam and Noun, with the former known to have the most productive breeding sites for *Simulium* vectors and contributing greatly to transmission of onchocerciasis in the area.^23^ The Mbam has many tributaries, of which the Kim and Nja Rivers are the main ones. Earlier studies indicated *Simulium sirbanum* and *Simulium squamosum* are the main vector species in this area.^24^

In this area, the main dry and rainy seasons run from December to March and from August to November, respectively. This is followed by a short light rainy season from April to June and a short dry season in July. The study was conducted in the rainy season from 7 October to 14 November 2019 and in the dry season from 16 January to 18 February 2020.

### Study design

This was an experimental study design using the standard HLC and a modified version for fly collection. The two techniques were compared in terms of the number of flies collected and their physiological status during the collection period.

### Standard and modified HLCs

In the standard HLC, the collector sat on a stool and rolled up his/her trousers to knee level, exposing the feet and lower legs. Flies that landed on them were caught using a mouth aspirator before they commenced blood feeding.

The modified HLCs were carried out in the same manner as the standard HLC. However, instead of exposing their lower limbs, the collectors wore trousers of blue, black or blue-black, corresponding to the three modified collection methods. The fabric was made of cotton material and the collectors wore their normal clothes before wearing the fabrics.

### Fly collection

Data from a previous entomological study conducted in this area^22^ were reviewed and two breeding sites on the Mbam and Nja were selected for blackfly collection. The two rivers are approximately 3 km apart, thus these breeding sites are likely within the same transmission zone. Along each breeding site, four catch points were identified 50 m apart from each other and located at equal distances linearly from the breeding site. These collection points were used to assess the efficacy of the standard HCL (sHCL) technique and the modified versions using blue trousers (Blu), black trousers (Bla) and blue-black trousers (Blu/Bla).

Collection of blackflies was conducted between 07:00 h and 17:00 h over four consecutive days per month. Two trained collectors ensured the collection interchangeably at each collection point, one collecting from 07:00 h to 12:00 h and the other from 12:00 h to 17:00 h. Rotation minimized any bias at the same collection point and also reduced fatigue. To further minimize bias due to the position of the collection point and the attractiveness of the collectors and their trousers, collectors were rotated on the different collection points following a 4×4 Latin square. The collectors used a locally made aspirator labelled with the collection site, the HCL technique and the hour of collection.

### Fly storage and dissection

Caught flies were transported hourly in the aspirators to the field laboratory (by the collector who was not catching at that moment) for specimen count and dissection as described by Davies and Crosskey.^25^ In brief, the flies were anaesthetized using vapour of an ether solution and drowned in lightly soaped distilled water (detergent) in a petri dish to ease fly counting. After counting, the flies were dissected under a dissecting microscope at 40× magnification in normal saline to identify those that were parous (presence/absence of follicular relicts and elasticity of the ovaries, the Malpighian tubules for opaqueness and the abundance of abdominal fat). The abdomen, thorax and head of those identified as parous were further dissected for OV infections (stages L1, L2 and L3). Identified stages of infection were counted and all information was recorded on a paper data collection sheet. A very small proportion of the flies collected (approximately 0.5%) were damaged during transportation to the field laboratory and could not be dissected.

### Data analysis

Statistical analyses were conducted using R version 4.3.0 (R Foundation for Statistical Computing, Vienna, Austria).^26^ Hourly rates of flies caught were analysed using a negative binomial generalised linear model, which is appropriate for overdispersed count data, to explore associations between flies caught and season, as well as the trapping method. Results from both the univariate and multivariate models were presented.

The parous rate, the proportion of dissected flies that were classed as parous, and the daily biting rate, representing the number of flies caught on landing (as a proxy of bites received by an exposed person) during the day, were calculated. This was used to calculate the monthly biting rate (MBR) as the total number of blackflies collected in the month divided by the number of days of capture during that month, multiplied by the number of days in the particular month. To explore the associations between the proportion of parous flies (outcome) and the method and season, a binomial generalised linear model was conducted using a 5% significance level.

Other entomological indices such as infection and infectivity rates were also calculated as the proportion of parous flies with L1, L2 or L3 and parous flies with L3 (in the head), respectively. However, these rates are not compared in this article.

## Results

### Blackly collection and entomological indices

Combining all capture activities (sHLC and all modified HLCs), a total of 17 246 flies were caught for the entire 4-month study, including 10 757 (62.4%) in the rainy season and 6489 (37.6%) in the dry season. Of those collected during the rainy season, 10 708 (99.5%) were dissected and 381 (3.6%) were found parous, of which 2 (0.5%) harboured L1 and L2 parasites indistinguishable as OV. No L3 infective larvae were found (Table 1). Of the 6489 collected during the dry season, 6483 (99.9%) were dissected and 292 (4.5%) were identified as parous. Among those parous flies, 3 (1.0%) were L1, L2 and L3 larval stage OV parasites, with one of the three harbouring L3 infective larvae in the head. October had the highest MBR of 43 198.5 bites/person/month and February the least (17 787 bites/person/month).

**Table 1.** Collection and dissection of blackflies caught during the rainy and dry seasons by all four HLC techniques

HLC technique	Blackflies caught, n	Blackflies dissected, n	Parous, n (%)	Infected, n (%)	Infective, n (%)
Rainy season (October–November)					
sHLC	3187	3138	111 (3.5)	1 (0.9)	0 (0.0)
Blu	2412	2412	102 (4.2)	0 (0.0)	0 (0.0)
Bla	3330	3330	105 (3.2)	1 (1.0)	0 (0.0)
Blu/Bla	1828	1828	63 (3.4)	0 (0.0)	0 (0.0)
Total	10 757	10 708	381 (3.6)	2 (0.5)	0 (0.0)
Dry season (January–February)					
sHLC	1943	1943	78 (4.0)	2 (2.6)	1 (1.3)
Blu	1305	1300	47 (3.6)	0 (0.0)	0 (0.0)
Bla	1818	1817	87 (4.8)	1 (1.1)	0 (0.0)
Blu/Bla	1423	1423	80 (5.6)	0 (0.0)	0 (0.0)
Total	6489	6483	292 (4.5)	3 (1.0)	1 (0.3)

Table 1 provides details of fly collection and entomological indicators among the different HCL collection versions.

### Comparing collections between standard and modified HLC versions

Combining all seasons, sHLC caught a total of 5130 (29.7%) flies, Bla HLC captured 5148 (29.9%), Blu HLC caught 3717 (21.6%) and Blu/Bla HLC captured 3251 (18.9%). As shown in Table 2, the negative binomial models showed a significant association between trapping methods and fly catches (p<0.01) and between season and fly catches (p<0.01). The blue and blue-black trousers caught significantly fewer flies than the sHLC method. There was no significant difference between the Bla HLC method and the sHLC method. More flies were captured during the rainy season than the dry season. Results from the univariate model and the multivariate model including season were very similar.

**Table 2.** Comparison of alternative methods versus sHLC

Method	Univariate model, rate ratio (95% CI)	p-Value	Multivariable model, rate ratio (95% CI)	p-Value
sHLC (ref)		<0.01		<0.01
Blu	0.72 (0.60 to 0.88)		0.71 (0.59 to 0.86)	
Bla	1.00 (0.83 to 1.21)		0.99 (0.82 to 1.19)	
Blu/Bla	0.63 (0.52 to 0.77)		0.65 (0.54 to 0.78)	
Season: rainy (ref=dry)	1.66 (1.45 to 1.90)	<0.01	1.64 (1.43 to 1.87)	<0.01

There was no significant association between the method used and the proportions of parous flies captured (p=0.57). However, the proportion of parous flies was lower during the rainy season compared with the dry season (OR 0.79 [95% confidence interval {CI} 0.68 to 0.92]). Due to low numbers of infected (n=5) and infective (n=1) flies, the mean proportions could not be compared statistically between techniques or seasons.

## Discussion and conclusion

This study demonstrates a viable method to protect collectors while effectively collecting blackflies. Black-coloured trousers performed equally well (no significant difference) as the sHLC technique, catching a similar number of flies that were of comparable physiological status. Its use is also feasible and sustainable, as these fabrics are readily available in local markets at low cost, making it possible to customise a functional protective suit for any person (approximately $20) involved in fly collection. Other studies have also reported the performance of black colour being a better visual attractant for anthropophilic flies, including blackflies.^27,28^ Furthermore, the similarity of physiological status among flies captured with this method enables calculation of transmission indices, including annual transmissions potential, which is recommended as an alternative indicator for assessing transmission when captured fly numbers are too low for pool-screening analysis.

Variations in seasonal collection of flies are related to ecological and environmental conditions at each breeding site. In some areas, breeding sites are flooded during the rainy season, leading to low numbers of flies, while fly density increases when the water level recedes during the dry season. In others, the inverse is true, with high water levels during the rainy season creating ideal conditions and inadequate water levels during the dry season. In this study, most of the flies were caught during the rainy season, adding to the existing knowledge of this period being considered as the peak transmission period where the fly population is much higher compared with the dry season.^29–31^ However, it is highlighted that there were still sizable fly catches during the dry season, indicating year-round conditions suitable for blackfly breeding and onchocerciasis transmission within the Massangam HD.

These results demonstrate that black-colour trousers present a safer, more ethical alternative that can effectively replace the current HLC method, especially in this era of elimination where all countries need to collect approximately 6000 blackflies per transmission zone for entomological evaluations. Furthermore, with the WHO 2030 roadmap increasing the number of countries verified as having interrupted transmission,^8^ a decrease (by 26.9%) in the number of people treated for onchocerciasis in 2020 compared with 2019 and more than 240 million people still at risk,^32^ the need for safer collection techniques cannot be overemphasized.

Despite the potential impact of the study providing a breakthrough in long-standing ethical concerns, this study was conducted in just one transmission area. Thus further studies are needed to confirm the generalizability of the results of this modified HLC in different ecological zones and transmission environments with well-characterised cytotaxonomically identified species.

## Data Availability

All relevant data are within the manuscript.
